# Editorial: Novel molecular mechanisms and clinical strategies in solid tumor recurrence and metastasis: from bench to bedside

**DOI:** 10.3389/fmed.2024.1364666

**Published:** 2024-02-13

**Authors:** Hangcheng Fu

**Affiliations:** Department of Urology, University of Louisville School of Medicine, Louisville, KY, United States

**Keywords:** renal cell carcinoma, solid tumor, oligometastase, breast cancer, molecular marker

In the realm of oncology, the pursuit of understanding and treating various forms of cancer continually evolves, bringing to light novel insights and therapeutic strategies. Recent studies in the fields of breast cancer, renal cell carcinoma (RCC), and small cell lung cancer (SCLC) exemplify this progress, highlighting the importance of interdisciplinary research and personalized treatment approaches.

## Deciphering molecular mechanisms of acute kidney injury in RCC

The study by Wang et al., “*Identification of AKI Signatures and Classification Patterns in ccRCC Based on Machine Learning*,” ventures into the intricate relationship between Acute Kidney Injury (AKI) and clear cell renal cell carcinoma (ccRCC). Previous study revealed that compromised CKD after nephrectomy was associated with worsening overall survival however the group of patients are of great heterogeneity (1). Utilizing public databases and machine learning algorithms, the research identifies seven novel AKI biomarkers. These biomarkers not only aid in early AKI detection but also distinguish between two subtypes of ccRCC with different prognoses and immune characteristics. This study underscores the power of machine learning in unraveling complex biological relationships, offering a significant leap in personalized cancer treatment. It can help us to better understanding the mechanism behind the renal cell carcinoma pathology and AKI induced CKD.

## Advancing SCLC treatment with combined therapies in solid tumors

In the realm of lung cancer, the study “*Efficacy and Safety of Immune Checkpoint Inhibitors Combined with Chemotherapy as First-line Treatment for Extensive-stage Small Cell Lung Cancer*” is a meta-analysis based on mixed-effect models (Zheng et al.). It shows that combining immune checkpoint inhibitors (ICIs) with chemotherapy significantly prolongs survival in patients with extensive-stage SCLC. This combination, however, does not markedly improve the objective response rate or disease control rate and raises the incidence of immune-related adverse events. These findings spotlight the need for a nuanced understanding of treatment benefits vs. risks, informing clinical decision-making in SCLC management.

## Radiotherapy in oligometastases: a paradigm shift

Kao et al. shifts the focus to a less explored area in cancer treatment—oligometastases. They defined <5 metastatic lesions in the radiology finding as oligometastases. The study reports encouraging outcomes from comprehensive involved site radiotherapy, often in combination with systemic therapy. With a significant proportion of patients achieving long-term remissions and high rates of local control, the study advocates for a potential paradigm shift in treating metastatic cancers. It also highlights the predominance of distant failures, suggesting a tailored approach to managing minimal residual disease.

## Breast cancer: a molecular culprit

In breast cancer research, the study “*Association Between Endocrine Therapy and Cognitive Decline in Breast Cancer Based on Propensity Score Matching*” by Yin et al. addresses a crucial aspect of cancer treatment—the balance between therapeutic efficacy and side effects. The research reveals a concerning association between prolonged endocrine therapy in breast cancer patients and cognitive impairment. By utilizing propensity score matching, the study ensures a rigorous evaluation of this relationship, adding a crucial dimension to patient care in breast cancer treatment.

In addition, Lian et al. delves into the molecular intricacies of breast cancer. The over-expression of SLC31A1, a gene associated with copper transport and cuproptosis, is linked to poor prognosis and increased immune cell infiltration in breast cancer. This study not only opens new avenues in understanding breast cancer progression but also posits SLC31A1 as a potential target for novel therapeutic strategies. Miao et al. highlights the role of the DYNLT1 gene in breast cancer. This study demonstrates that DYNLT1 is overexpressed in breast cancer and is associated with poor relapse-free survival. It further explores the mechanisms by which DYNLT1 influences tumor cell proliferation and metastasis, offering new insights into potential targets for therapy and prognostication in breast cancer.

## Future perspectives

These studies collectively signify a monumental shift in cancer research. They emphasize the importance of integrating findings from molecular biology, machine learning, and epidemiology into clinical practice. The challenge ahead lies in translating these insights into tangible benefits for patients, balancing treatment efficacy with quality of life.

Moreover, these studies underscore the importance of considering the holistic impact of cancer treatment. From cognitive effects in breast cancer patients undergoing endocrine therapy to the challenges of managing adverse events in SCLC treatment, a patient-centric approach is paramount.

As cancer research continues to advance, a multidisciplinary approach becomes increasingly vital. The integration of data science, molecular biology, and patient-centered clinical research holds the key to unraveling the complexities of cancer. The future of oncology lies in the ability to personalize treatment, minimize side effects, and improve overall patient outcomes.

## Author contributions

HF: Writing—original draft, Writing—review & editing.
